# Biomass Production Potential in a River under Climate Change Scenarios

**DOI:** 10.1021/acs.est.1c03211

**Published:** 2021-08-03

**Authors:** Paulina Orlińska-Woźniak, Ewa Szalińska, Ewa Jakusik, Damian Bojanowski, Paweł Wilk

**Affiliations:** † Institute of Meteorology and Water Management, National Research Institute, Podleśna 61, Warsaw 01-673, Poland; ‡ Faculty of Geology, Geophysics and Environmental Protection, AGH University of Science and Technology, A. Mickiewicza Av. 30, Krakow 30-059, Poland

**Keywords:** biomass production potential, chlorophyll “a”, river basin, Macromodel DNS/SWAT

## Abstract

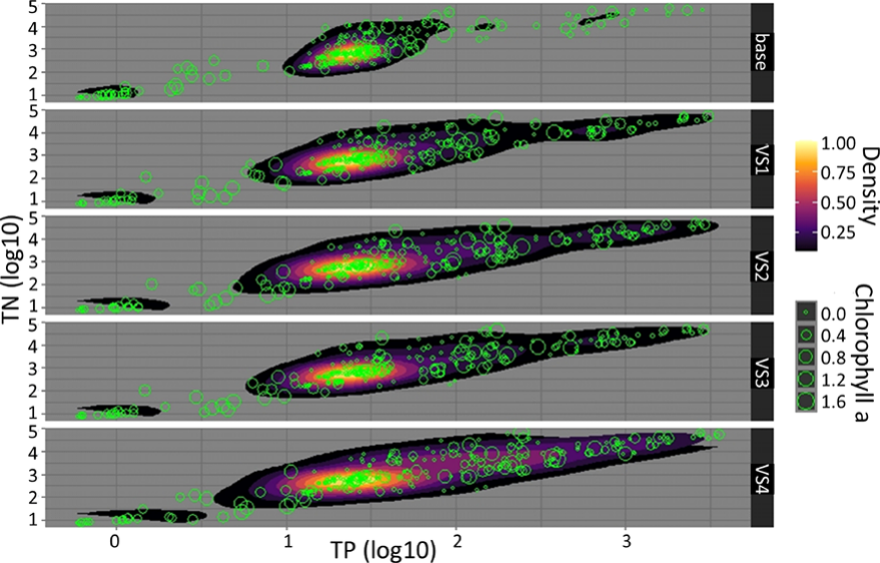

Excessive production of biomass, in times of intensification of agriculture and climate change, is again becoming one of the biggest environmental issues. Identification of sources and effects of this phenomenon in a river catchment in the space–time continuum has been supported by advanced environmental modules combined on a digital platform (Macromodel DNS/SWAT). This tool enabled the simulation of nutrient loads and chlorophyll “a” for the Nielba River catchment (central-western Poland) for the biomass production potential (defined here as a TN:TP ratio) analysis. Major differences have been observed between sections of the Nielba River with low biomass production in the upper part, controlled by TN:TP ratios over 65, and high chlorophyll “a” concentrations in the lower part, affected by biomass transport for the flow-through lakes. Under the long and short-term RCP4.5 and RCP8.5 climate change scenarios, this pattern will be emphasized. The obtained results showed that unfavorable biomass production potential will be maintained in the upper riverine sections due to a further increase in phosphorus loads induced by precipitation growth. Precipitation alone will increase biomass production, while precipitation combined with temperature can even enhance this production in the existing hot spots.

## Abstract 2

Modeling approach assists in current and future water eutrophication analysis in a river under climate change scenarios.

## 1 Introduction

The biomass overproduction problem has been attracting the attention of the scientific community for several decades,^1−5^ which resulted in numerous studies, mainly on nitrogen and phosphorus compounds, and their mutual relationship implicating eutrophication processes.^6−9^ Along with a better understanding of causes and effects behind this phenomenon, development of environmental models took place, enabling simulation of current and future changes in aquatic nutrient issues.^10−13^ However, these modeling efforts have been mainly focused on lakes and reservoirs,^14−20^ while neglecting, to a large extent, eutrophication processes in riverine ecosystems.^21−23^ Numerical simulations of nutrient loads and concentrations of chlorophyll “a” in flowing surface waters are still very rare. Currently, the main source of information on this topic is comprehensive research conducted on river basins in South Korea,^17,19,23−30^ and individual publications from other regions of the world.^21,31−33^ These studies clearly show that one of the promising methods to understand complex interactions influencing biomass production in rivers may be the use of environmental models, such as ANNs,^24^ QUALKO2,^34^ Hidden Markov Model (MHMM),^29^ or SWAT.^33^ Moreover, the combined capabilities of different models are also increasingly used^35,36^ as well as artificial neural networks^37^ or machine learning methods.^38^ Nevertheless, the influence of factors, such as surface runoff or future changes in temperature and precipitation, is rarely taken into account. Meanwhile, surface runoff is one of the most important processes causing the migration of pollutants from the basin land phase to the river, and future changes in temperature and precipitation will have a major impact on this process. Therefore, there is a justified risk of altering biomass production in river basins.^39,40^

Concomitantly occuring anthropogenic and climate pressures exerted on river system are increasing,^31,41−43^ and therefore a so-called “new wave of eutrophication” is frequently reported.^44,45^ This phenomenon is mainly caused by human activity, more specifically by growing demand for food production, leading to intensification of agriculture, and an increase in nitrogen and phosphorus loads from nonpoint sources. This also includes a dynamic development of urbanized areas and an increase in industrial and municipal wastewater discharges. Moreover, it is due to the acceleration of climate change,^46−48^ defined as the possibility of biomass production, described by selected factors (e.g., nitrogen and phosphorus ratio, solar radiation, temperature, etc.). Local and regional environmental variables (e.g., limnological, morphometric, and land use) are also important. While the anthropogenic factors influencing biomass production are well described, the impact of climate change is still ambiguous.^24,29,49^ The processes contributing toward eutrophication in a river can be distinguished between the land and river bed phases. In the land phase, a precipitation increase in selected months of the year will contribute to intensification of the surface runoff and pollutant wash-out^39,40^ and their subsequent transport into a river. This process may be partially limited by an increase in temperature, leading to, among the others, an extension of the vegetation period.^30^ This will result in a longer plant cover occurrence that will enhance nutrient uptake from soils and offer protection against erosion caused by rainfalls.^50,51^ In the river bed phase, however, climate change may contribute to the extension of the period with conditions favorable for the acceleration of in situ biomass production (e.g., temperature and solar radiation).^52,53^

The aim of this research was to trace current and future biomass production in a river, resulting from nitrogen and phosphorus discharges from its basin. For the purposes of this article, the concept of “biomass production potential” has been defined and related to the nitrogen and phosphorus ratios. Moreover, the impact of climate change on the eutrophication potential of the river, using an advanced environmental model was used, based on these nitrogen and phosphorus load ratios. For this purpose, a small but strongly agricultural basin of the Nielba River in central-western Poland was selected, featuring three flow-through lakes on the main watercourse. The numerical model of the Nielba River basin was created in the Macromodel DNS/SWAT (Discharge-Nutrient-See/Soil & Water Assessment Tool) digital platform and its chlorophyll “a” module (CHLA) and was used to track the biomass production potential in time and space along the entire river continuum. Ultimately, the question on present chlorophyll “a” concentrations in the specified calculation profiles of the analyzed river and their changes in the near and distant future have been answered. Moreover, mathematical description of these changes has been applied as a first stage of the biomass production potential analysis. The importance of our method lies in the simplicity of our approach, which can be applied without additional complicated tools for the TN, TP, and chlorophyll “a” data analysis to assess future changes in biomass production.

## 2 Materials and Methods

### 2.1 Study Area

**Figure 1:**
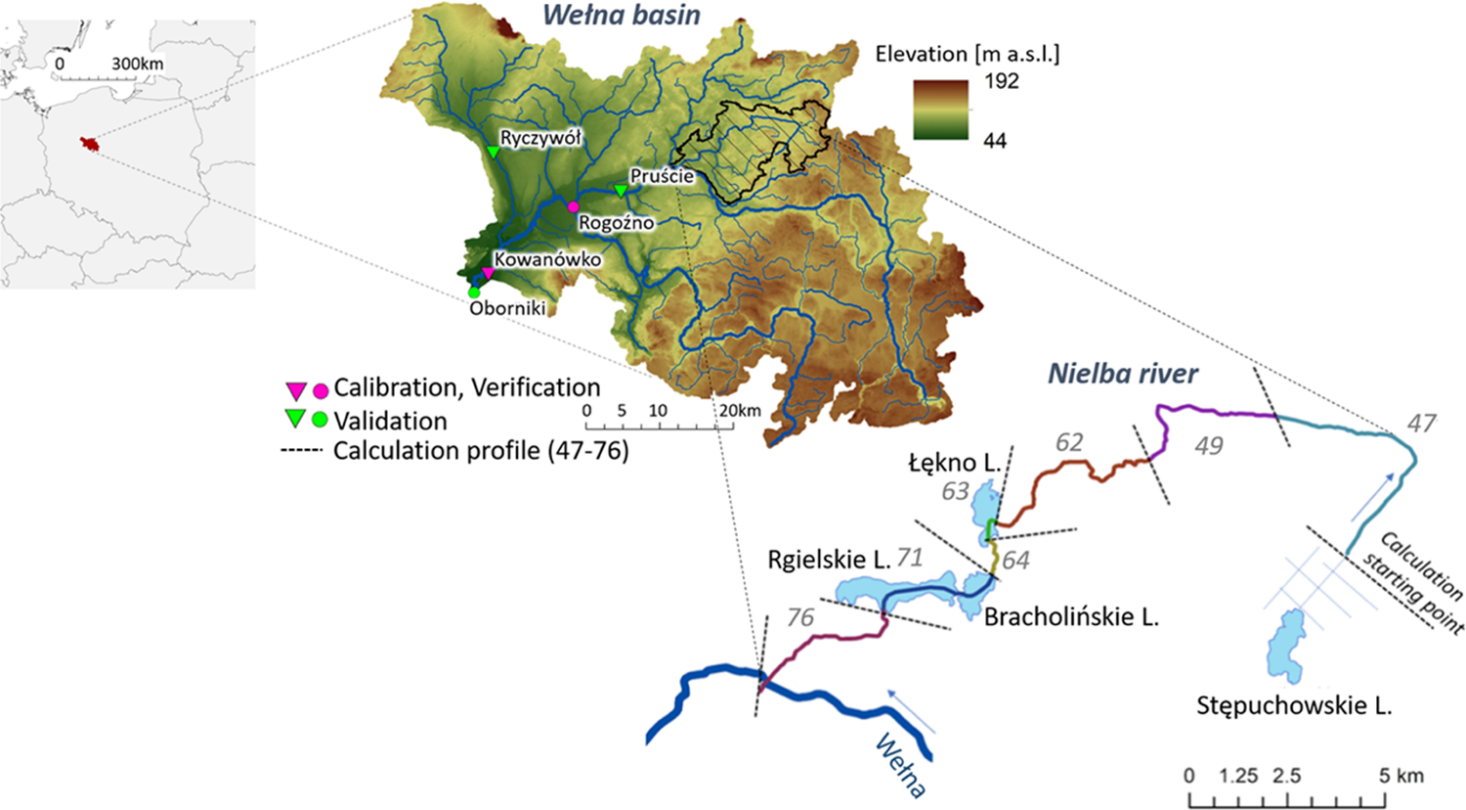
Location of the Nielba River along with the division into sub-basins (47, 49, 62, 63, 64, 71, and 76) including flow-through lakes.

The Nielba River basin (158.6 km^2^) (Figure 1) is located in the central-western part of Poland and belongs to the Wełna river basin (right-bank tributary).^54−57^ It is an area dominated by intensive agriculture and very prone to eutrophication. The calculation starting point of the Nielba River, defined as the source, is hydraulically connected by a set of man-made canals to the Lake Stępuchowskie. The river, 30.7 km long, has been divided into seven sub-basins (47, 49, 62, 63, 64, 71, and 76; Figure 1), with three flow lakes in its lower part. Their detailed description together with information on the use of the basin area and its hydrological and meteorological conditions are described in the Supporting InformationCh1 and Figure S1. The Nielba river water quality context is presented in the Supporting Information Ch2.

### 2.2 Nutrient and Chlorophyll “a” Assessments for Pilot Basin

**Figure 2:**
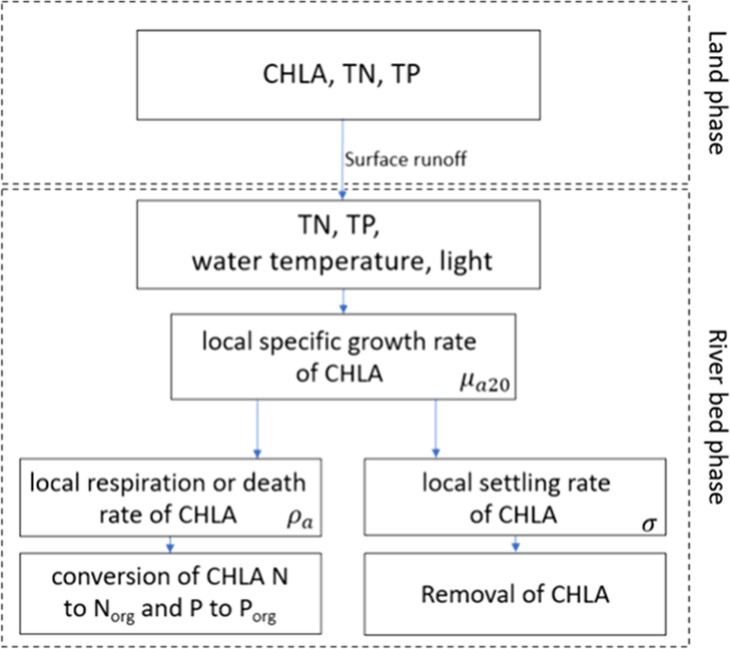
CHLA module in the Macromodel DNS/SWAT.

To estimate the concentration of chlorophyll “a” (CHLA), in selected calculation profiles of the analyzed river, the assumption that the concentration of CHLA is to be directly proportional to the concentration of phytoplankton algal biomass was used,^6^ which became the basis for the basin models^58^ and research on biomass production.^59−61^ The SWAT model and the CHLA simulation module were used for the analyses (Figure 2), and the method used in this tool is described in the Supporting Information Ch2. The model development and calibration for the Nielba River Basin are described in the Supporting Information Ch3. The SWAT model allows us to simulate the TN and TP loads and CHLA with a daily time step for the opening (in) and closing (out) calculation profiles of each designated basin. Due to the fact that the concentration of CHLA is largely an effect caused by the loads TN and TP and their interdependence (TN:TP ratio), therefore, loads of TN and TP (in) and CHLA (out) have been simulated in the current study.

### 2.3 Climate Scenarios

**Figure 3:**
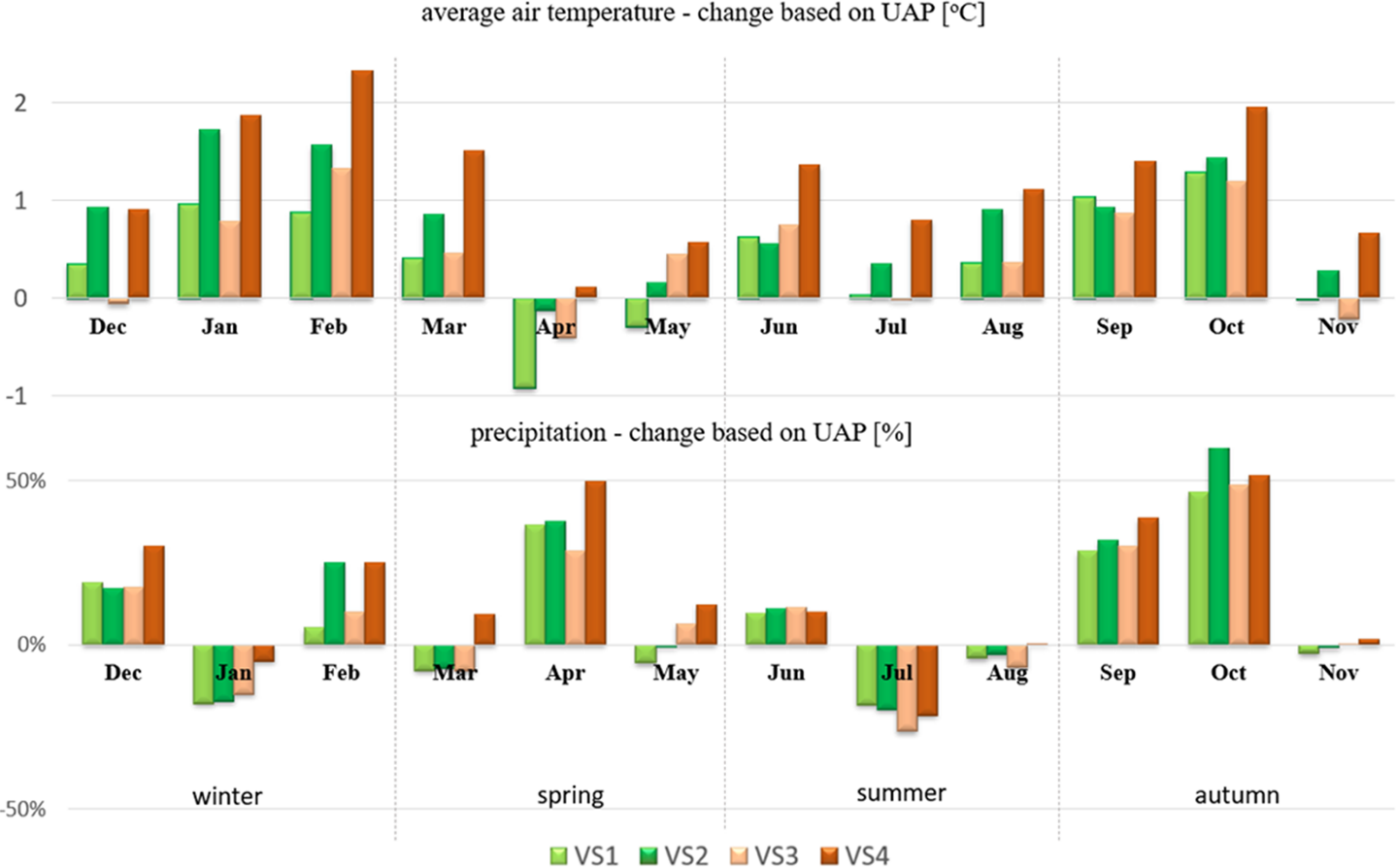
Changes in average monthly temperature and precipitation values adopted for the particular climate scenarios (based on the UAP project).

Used for the analyses, the UAP (Urban Adaptation Plans) project^62,63^ predictions have been based on the data from the Euro-CORDEX, Regional climate models (RCM), and the Global Climate Models (GCM) on a regular grid with a resolution of 0.11° (approx. 125 km).^64−66^ Data from the Poznań-Ławica synoptic station (52.416885, 16.834444) has been used, which is located 48.5 km away from the Nielba River basin, and there is a 22 m difference in altitude between both locations. The statistical postprocessing (downscaling)^67,68^ was performed using the tools available in the R environment.^69^ The climate condition analysis in the UAP project covered the moderate (RCP4.5) and extrapolative (RCP8.5) scenarios and two future time horizons: a short-term perspective (average of the 2026–2035 time period) and a long-term perspective (average of the 2046–2055 time period).^70−72^ Therefore, four climate variant scenarios, with a monthly time step, were prepared to combine the RCP predictions and adopted time horizons: VS1 (RCP4.5: 2026–2035), VS2 (RCP4.5: 2046–2055), VS3 (RCP8.5: 2026–2035), and VS4 (RCP8.5: 2046–2055) (Figure 3).

**Figure 4:**
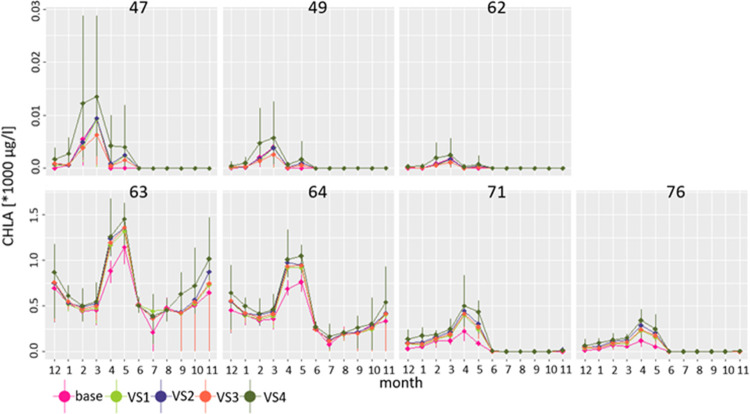
Monthly average chlorophyll “a” (CHLA) concentrations (*1000 μg/L) for the Nielba River sub-basins under the baseline and climate change scenarios (VS1–VS4).

### 2.4 Results Processing

**Figure 5:**
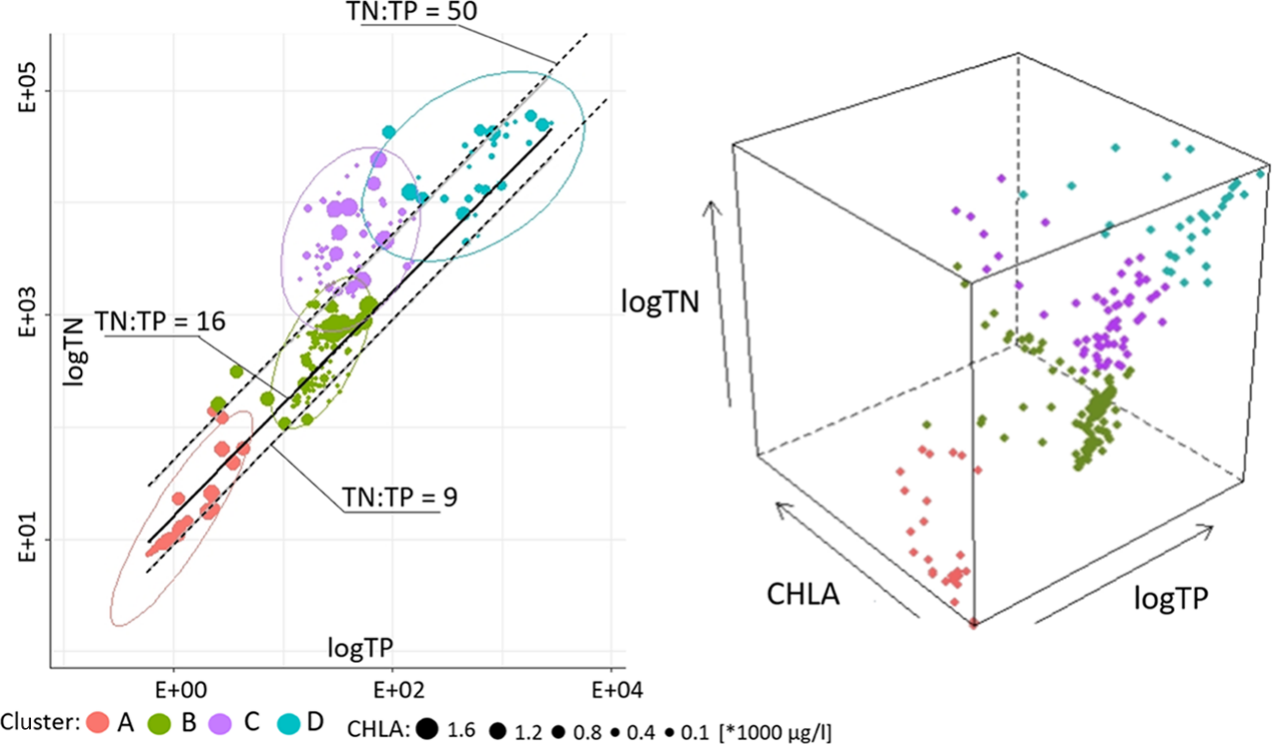
2D and 3D cluster distribution of the Nielba River for the baseline scenario.

**Figure 6:**
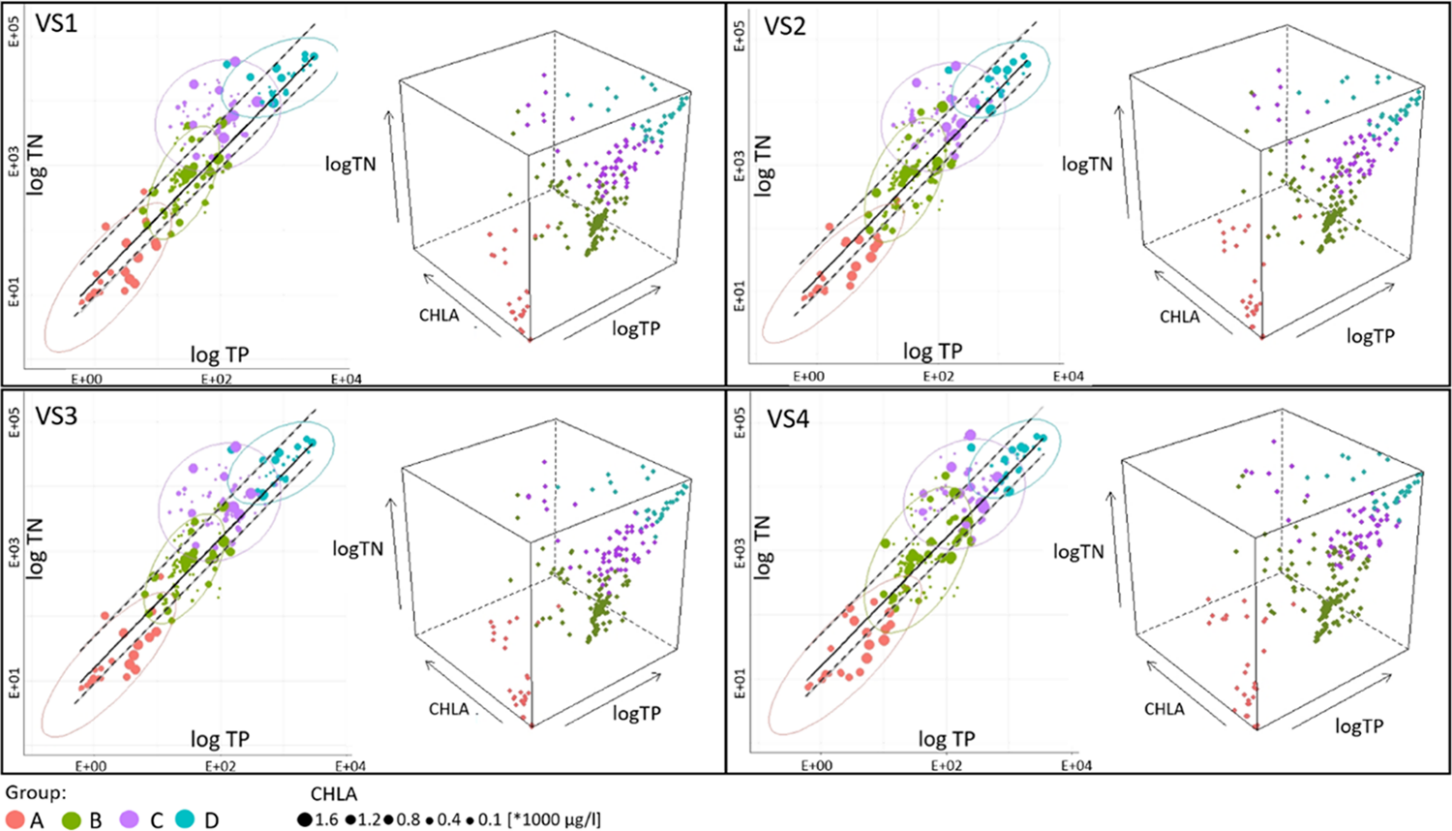
2D and 3D cluster distribution of the Nielba River under the variant scenarios.

To discuss the current and future biomass production issues in the Nielba River, the obtained modeling results have been presented in a form of 2D scatter plots (Figures 5 and 6). In these plots, TN and TP loads have been displayed for the entire Nielba River data set. Moreover, the resultant set of points has been size-coded to express the chlorophyll “a” concentrations and color-coded to show their affiliation to one of the four result groups (A–D). Division of the points into these groups has been performed with use of cluster analysis based on the sum of squared euclidean distance. The cluster analysis and determination of the 95% confidence ellipses around each group have been performed with the R software. To assess the most favorable conditions for the biomass development, the lines signifying TN:TP ratios 9, 16, and 50 have also been displayed in the figures. To track the impact of the variant scenarios and subsequent changes for the combination of all the three discussed variables (TN, TP, and CHLA), 3D scatter plots (Figures 5 and 6) have also been included. Division of the points into the 3D clouds (A–D) followed the previously applied cluster analysis. To express the extent of future changes under each variant scenario, 95% confidence 3D ellipsoid volumes have been calculated for each cloud (Table 3). Moreover, the absolute changes for each variable (TN, TP, and CHLA) have been given for each cluster/cloud.

## 3 Results

### 3.1 Nutrient Simulations

The average monthly nutrient load predictions in the Macromodel DNS/SWAT for the Nielba River sub-basins for 2005–2007 have been presented in Table 1 and in the Mendeley Data.^73^ The total nitrogen loads (TN) constantly increased from the source of the river (sub-basin 47, Figure S2) to the inflow of the Łękno Lake (sub-basin 63), in total, by over 4900 kg/month (i.e., 98%). A similar pattern has been displayed by total phosphorus (TP), with loads increasing by 130 kg/month (180%) in the same section of the river. As for the ratio of both nutrient loads (TN:TP), it varied from 65 in sub-basin 49 up to 76 in sub-basin 62.

**Table 1:** Average Monthly Nutrient Loads (kg/month), TN:TP Ratios, and Chlorophyll “a” Concentrations (μg/L) for the Nielba River (Baseline Scenario) with the Coefficient of Variation Values (CV) in Brackets

sub-basin	TN	TP	TN:TP	CHLA
47	4983 (133%)	81 (164%)	75 (85%)	1.3 (230%)
49	6095 (135%)	163 (173%)	65 (82%)	0.5 (236%)
62	9420 (135%)	226 (179%)	76 (98%)	0.2 (239%)
63	9886 (130%)	210 (162%)	75 (93%)	578.8 (41%)
64	61 (127%)	3 (118%)	18 (56%)	364.2 (53%)
71	2245 (133%)	73 (169%)	51 (117%)	54.2 (133%)
76	2504 (124%)	78 (151%)	45 (119%)	29.3 (135%)

The presence of the Łękno Lake drastically changes the nutrient dynamic in the Nielba River. The decrease by 99 and 96% for both TN and TP, respectively, is visible at the opening calculation profile of sub-basin 64, directly downstream from the lake. The change in the nutrient loads also brings a decrease in the TN:TP ratio, which for this sub-basin, is 18. Along with the subsequent river course, another increase in TN and TP loads is visible, despite the presence of the two other lakes (Bracholińskie and Rgielskie). However, the load values reached only 2504 and 78 hg/month for TN and TP, respectively (Table 1).

Apart from the spatial variability of nutrient loads among the Nielba River sub-basins, the temporal one shall also be noticed. The pronounced monthly variabilities of TN and TP loads, expressed as coefficients of variation (CV), ranged from 124% up to 135% and from 118% up to 179% for TN and TP, respectively (Table 1). The monthly load distribution (Figure S2) showed elevated values of nutrient loads in the winter and early spring months, with the maximum values in March (37,983 and 1345 kg/month for TN and TP, respectively, in sub-basin 62) and the minimum ones in August (454 and 38 kg/month, respectively, for TN and TP, respectively, in sub-basin 47). Large fluctuations of nutrient loads during the year directly translated into the TN:TP ratio with a variability range from 9 in sub-basin 64 in May to even 253 in December in sub-basin 62.

### 3.2 Chlorophyll “a” Simulations

The simulated chlorophyll “a” (CHLA) concentrations were very low in the upper part of the Nielba River with a maximum value of 1.3 μg/L for sub-basin 47 (Figure 4 and in the Mendeley Data^73^). Moreover, a decreasing trend along the river course toward the Łękno Lake has been observed, until the value reached 0.2 μg/L at the river outflow to the lake (sub-basin 62). Simultaneously, with the depletion of nutrients due to rapid eutrophication in the lake, the increase in CHLA concentration reaching almost 580 μg/L at the outlet of the Łękno Lake was observed. The CHLA concentrations remained at a high level also in the riverine section connecting the Łękno Lake, with the Bracholińskie Lake (sub-basin 64, over 364 μg/L). Downstream from the last lake on the river course (Rgielskie Lake, sub-basin 71), a noticeable decrease in CHLA values was visible, and by the last urbanized section of the Nielba River basin (sub-basin 76), the CHLA value was reduced even further down to 29.3 μg/L.

Despite the small average values, CHLA concentrations for the upper part of the river displayed very high variability, with the CV values in a range of 230–239%. This phenomenon is caused by the chlorophyll increase during late winter and early spring months (February and March), with an average value of 9.28 μg/L in March at sub-basin 47 (Figure 4). Downstream from the Łękno Lake, a difference in the CHLA monthly distribution can be observed, with extreme values shifted toward the late spring months (April and May). Moreover, a different pattern can also be observed in sub-basins 63 and 64, with the summer CHLA concentration decrease followed by another increase during the autumnal months. Meanwhile, in sub-basins 71 and 76, CHLA concentrations remained low during the autumn period after the summer decrease.

### 3.3 Climate Change Simulations

The four adopted variant scenarios allowed tracking the impact of climate changes under the RCP4.5 and RCP8.5 predictions for the modeled parameters in the two time horizons.^73^ The forecasted changes of meteorological conditions in the Nielba River basin will affect both nitrogen and phosphorus loads, especially under the RCP8.5 long-term scenario (VS4). Generally, the average TN and TP loads will increase along the entire length of the studied river. However, the highest changes in TN should be expected in the last two sub-basins of the river (71 and 76) for VS1–VS3 scenarios by 15–21% of the baseline scenario values and by 80% is sub-basin 64 for the VS4 scenario (Table 2). As for the TP loads, a dramatic increase will be observed in the first sub-basin of the studied area by 111–193% for all the adopted scenarios. Moreover, this increase in TP values is again predicted for the last two sub-basins (71 and 76). These pronounced changes in nutrient loads will naturally influence the TN:TP ratio values. Generally, a decrease in these ratios can be expected in a range of 15–59% for all the variant predictions. However, insofar, changes in the first three sub-basins (47–62) will still keep the level of TN:TP ratios generally above 50, whereas the changes in the last two sub-basins (77 and 76) will provoke their drop, even down to 21 under VS4.

**Table 2:** Average Monthly Nutrient Loads (kg/month), TN:TP Ratios, and Chlorophyll “a” Concentrations (μg/L) for the Nielba River under the Variant Climate Change Scenarios (VS1–VS4)^a^

	VS1				VS2				VS3				VS4			
	TN	TP	TN:TP	CHLA	TN	TP	TN:TP	CHLA	TN	TP	TN:TP	CHLA	TN	TP	TN:TP	CHLA
47	5366 (8%)	184 (127%)	59 (−22%)	1.44 (12%)	5463 (10%)	193 (137%)	55 (−26%)	1.59 (24%)	5435 (9%)	171 (111%)	54 (−28%)	1.14 (−11%)	7115 (43%)	238 (193%)	58 (−23%)	3.20 (148%)
49	6651 (9%)	288 (76%)	54 (−15%)	0.57 (9%)	6834 (12%)	315 (93%)	48 (−25%)	0.62 (18%)	6692 (10%)	275 (68%)	50 (−22%)	0.43 (−17%)	8713 (43%)	385 (135%)	47 (−26%)	1.20 (128%)
62	10,110 (7%)	332 (47%)	62 (−17%)	0.24 (10%)	10,157 (8%)	362 (60%)	56 (−26%)	0.26 (19%)	10,061 (7%)	314 (39%)	58 (−23%)	0.18 (−16%)	12,714 (35%)	440 (94%)	52 (−31%)	0.52 (133%)
63	10,926 (11%)	339 (61%)	62 (−17%)	648.88 (12%)	10,935 (11%)	371 (76%)	55 (−26%)	675.59 (17%)	10,847 (10%)	321 (53%)	57 (−23%)	656.80 (13%)	13,728 (39%)	455 (116%)	50 (−33%)	747.85 (29%)
64	67 (11%)	4 (44%)	15 (−13%)	412.06 (13%)	62 (2%)	6 (92%)	12 (−30%)	435.98 (20%)	66 (8%)	4 (44%)	14 (−22%)	423.90 (16%)	110 (80%)	7 (128%)	15 (−16%)	487.35 (34%)
71	2620 (17%)	118 (62%)	38 (−24%)	93.58 (73%)	2643 (18%)	146 (101%)	28 (−44%)	112.52 (108%)	2588 (15%)	129 (77%)	35 (−30%)	100.64 (86%)	3306 (47%)	191 (162%)	20 (−59%)	143.15 (164%)
76	2956 (18%)	125 (60%)	36 (−20%)	54.54 (86%)	3025 (21%)	154 (97%)	27 (−38%)	69.72 (138%	2910 (16%)	135 (73%)	33 (−26%)	59.70 (104%)	3735 (49%)	207 (165%)	20 (−53%)	87.31 (198%)

a Values in brackets signify changes in respect to the baseline scenario.

The described above changes of nutrient loads and their ratios will also affect chlorophyll “a” concentrations. However, different impacts of climate scenarios on CHLA values are observed. Under the RCP4.5 scenarios (VS1 and VS2), an increase in the CHLA concentrations is predicted by 9–24% of the baseline scenario values in the sub-basins 47–64 and by 73–138% in the sub-basins 71 and 76 (Table 2). Under the short-term RCP8.5 scenario (VS3), a decrease in CHLA values for the upper part of the Nielba River (sub-basins 47–62) was followed by a CHLA concentration increase, even up to 104% in the last sub-basin (76). As for the long-term predictions of the same scenario (VS4), a dramatic increase in the CHLA values can be expected (128–198%), except for sub-basins 63 and 64 (29–34%). However, it should be noted that for the upper part of the river (sub-basins 47–62), the CHLA concentrations will remain at a low level (<3.5 μg/L). The monthly distribution pattern of CHLA concentration (Figure 4) demonstrates that in the variant scenarios, CHLA will generally increase in significance in the late winter (February) and spring months (March–May) in all sub-basins. Meanwhile, in the remaining months of the year (especially in summer), CHLA concentrations will still remain at a relatively low level in each section.

The range of the variant scenarios’ impacts on the discussed variables (TN, TP, and CHLA) for the first time was estimated through the 2D and 3D analysis, which allowed for result interpretation on the spatial and temporal scale. This analysis included assessment of 95% confidence ellipses (2D) positioning in relation to the TN:TP ratio lines as well as the 95% confidence ellipsoid volumes (3D) for individual clusters (Table 3). Generally, a 2D cluster shift closer to the 9–50 lines suggests an increase in biomass production potential under future scenarios, while its scattering is a decrease. However, this will not necessarily result in a direct increase/decrease of biomass production, expressed by the chlorophyll “a” changes.

**Table 3:** Ellipsoid Volumes and TN, TP, and CHLA Changes for the Nielba River Clusters under Baseline and Variant Scenarios

cluster	scenario	ellipsoid volume	TN change	TP change	CHLA change
A	base	9			
	VS1	37 (407%)	56%	76%	15%
	VS2	36 (395%)	40%	140%	21%
	VS3	34 (382%)	48%	92%	17%
	VS4	43 (478%)	81%	205%	37%
					
B	base	124			
	VS1	411 (331%)	41%	26%	19%
	VS2	755 (609%)	76%	51%	28%
	VS3	452 (365%)	36%	39%	21%
	VS4	1609 (1298%)	156%	116%	51%
					
C	base	1747			
	VS1	5875 (336%)	30%	185%	27%
	VS2	6601 (377%)	28%	271%	37%
	VS3	5593 (320%)	30%	185%	31%
	VS4	9438 (540%)	72%	463%	52%
					
D	base	13,206			
	VS1	11,315 (85%)	–2%	58%	5%
	VS2	9973 (75%)	–2%	68%	12%
	VS3	10,775 (81%)	–1%	50%	11%
	VS4	10,997 (83%)	16%	92%	17%

The biggest changes in volume compared to the baseline scenario are to be expected for A–C ellipsoids. For the VS4 long time horizon scenario, these changes will even amount to over 1200%, while for cluster D, they will not exceed 85%. Generally, volume changes of all four ellipsoids will be primarily controlled by the TP loads, reaching even 465% (cluster C, VS4 scenario). Cluster C will be also characterized by the highest increase in biomass production (even by 52%), while again in cluster D, this increase will not exceed 17%.

## 4 Discussion

The existing methods of inland water eutrophication counteraction focus mainly on nutrient load determination and development of abatement measures in the affected basins.^31,45,74,75^ For these methods, modeling plays a complementary role, allowing not only prediction of spatial and temporal distribution of nutrients but also simulation of processes responsible for excessive biomass growth in surface waters. Although this problem is well discerned for lakes and reservoirs,^14−18^ attempts to verify and apply numerical models to analyze nutrient loads and chlorophyll “a” concentrations in flowing surface waters are still scarce.^27,28,75,76^ This is mainly due to the multiplicity and complexity of processes taking place and the limited amount of monitoring data in such systems.^17,29,45,38^ By using the chlorophyll “a” specific module (CHLA) incorporated in the Macromodel DNS/SWAT, we were able to track intricacies of spatial and temporal changes in biomass production in the river’s entire continuum from its source to the outlet cross-section.

### 4.1 Spatial Changes in Nutrient Loads and Chlorophyll “a” Concentrations

The spatial analysis revealed elevated values of the TN and TP loads already at the source of the river, which resulted mainly from the connection of the Nielba River source with the heavily polluted Stępuchowskie Lake through the system of canals (Figure S2). However, intensive agriculture and lack of proper wastewater management also play important roles in the nutrient delivery in this basin,^77^ which is clearly visible as a rapid increase in nutrient loads in the upper part of the river from the source to the discharge into the Łękno Lake is seen. The Nielba River displays a snow-rain hydrological regime that controls temporal variability of contaminant loads. Surface runoffs above 10^–5^ m^3^/s (Supporting Information Ch1), caused by snow melt, coincide with a spring fertilization period, resulting in the increase in nutrient delivery. It should also be noticed that the fertilization rate in this area is considered as the most intense in Poland, and therefore the local soils are extremely abundant in phosphorus compounds.^78^ Moreover, the small river flows, 0.25 m/s on average, in the upper part of the Nielba River do not guarantee proper dilution of nutrients and limit the self-purification processes.^79^ Despite such enrichment of the upper Nielba River waters with biogenic compounds, biomass production is practically negligible in this section of the river and does not exceed 1.3 μg/L of chlorophyll “a” as a mean monthly concentration. Therefore, this section can be considered re-oligotrophic.^80^ Since the TN:TP ratios vary in a range of 65–76 in this section of the river, the limiting role of phosphorus is very likely. Moreover, the dense shrub and tree canopy along the river banks reduces light access, further reducing the chlorophyll “a” development.^31,81,82^ The river flow effect is also considered to be a limiting factor for CHLA development.^29^

In the lower part of the river, the nutrient and biomass production patterns are controlled mainly by the lakes’ presence and the basin land use. The first lake on the river course, the Łękno Lake, serves as a receiver of nutrient discharges from the upper Nielba River and the local wastewater treatment plant. Therefore, it is well recognized for its highly hypertrophic waters.^56,83^ Rapid eutrophication in this lake causes an almost 98% decrease in TN and TP loads in the calculation profile located directly behind the lake (sub-basin 64). As for most of the lower Nielba River area, agriculture continues to be the main environmental pressure and is largely responsible for the increase in nutrients in this short section of the river (2.1 km) preceding the Bracholińskie Lake. Moreover, the presence of fish ponds in this area can be attributed as an additional source of pollution for this portion of the river.^84,85^ Despite the increase in nutrient loads, the TN:TP ratio remains at a more favorable level for the biomass production (around 50). This is directly reflected in the chlorophyll “a” concentrations, reaching over 360 μg/L, which corresponds to eutrophic conditions,^80^ causing the eutrophication phenomena. In the following section of the river, the two-lake system, Bracholińskie and Rgielskie, is connected by a 40 m-long stretch of the Nielba River (which in the past formed one lake)^86^ and affects the biomass production. The small and shallow Bracholińskie Lake receives high nutrient loads from the Nielba River and local sources of pollution, which results in hypereutrophic conditions.^87^ On the contrary, the depth of the Rgielskie Lake (over 5 m) clearly reduces the effectiveness of the eutrophication processes.^88−92^ Therefore, despite the still high nutrient loads discharged into this water body from the surface runoff and local point sources, the chlorophyll “a” concentration in the lake’s outlet is relatively low (54.2 μg/L). The last section of the studied river (sub-basin 76) differs significantly from the previous in terms of land use and is dominated by urban areas. However, the river channel remains still heavily bushed and forested. Even with TN:TP ratio values potentially promoting the eutrophication processes (below 50), rich delivery of nutrients from the municipal surface runoffs,^93,94^ and discharges from the local wastewater treatment plant, the biomass production is relatively low here (below 30 μg/L of chlorophyll “a”). The same range of chlorophyll “a” concentrations has been observed in many similar, in terms of length and land use, rivers across Europe.^31,95,96^

### 4.2 Biomass Production Potential

To estimate a potential for biomass changes in the particular reaches of the studied river, the TN:TP ratios between 9 and 50 have been adopted as favorable conditions for biomass development.^80,97−99^ Defined this way, biomass production potential was analyzed for individual identified 2D clusters along with significant features distinguishing it from the others. Cluster A contains outputs exclusively from sub-basin 64 with considerably large chlorophyll “a” concentrations, localized directly downstream from the Łękno Lake (sub-basin 63). In this cluster, the increase in the biomass potential when compared with the upstream sub-basin is noticeable (average TN:TP ratio of 15). This increase is most likely induced by the transport of biogens from the local hot spot (Łękno Lake) and to chlorophyll mobility characteristic for flowing surface waters.^100−102^ The most numerous cluster B comprises mostly summer/autumn modeling outputs from almost all the analyzed sub-basins. The majority of its records lie between TN:TP ratio 9 and 50 lines with the low chlorophyll “a” concentrations for the riverine sub-basins. The elevated chlorophyll “a” values for the Łękno Lake confirm all-year-round strongly eutrophic conditions in this sub-basin (63). In cluster C, covering exclusively winter/spring months, the biomass production potential based on the TN:TP ratios could be qualified as unfavorable (above 50). However, again, the impact of highly eutrophic conditions in the Łękno Lake (sub-basin 63) is evident, marking this cluster with infrequent high chlorophyll “a” concentrations. The rest of this cluster’s outputs indeed cover low biomass production from the riverine sub-basins. Cluster D encompases modeling outputs covering extremely high TN and TP loads. This cluster is almost entirely limited by the 9 and 50 TN:TP ratio lines that signify eutrophication favorable conditions. However, the sparse outputs with the elevated chlorophyll “a” concentrations in this cluster again belong to the lacustrine sub-basin (63). The rest of this cluster represents mostly low biomass production in the upper parts of the Nielba River. Similar relationships have been also observed in the other riverine systems.^24,49,103^

### 4.3 Temporal Changes under Climate Scenarios

The four adopted scenarios allowed us to track the impact of climate changes under the RCP4.5 and RCP8.5 forecasts in two time horizons (2026–2035 and 2046–2055). Foreseen changes in meteorological conditions in the Nielba River basin affected nitrogen and phosphorus loads and also chlorophyll “a” concentrations. In our approach, these changes have been traced following shifts in the designated ellipsoids volumes for each of the clusters.

In cluster A, containing data exclusively from the sub-basin located downstream from the Łękno Lake, the 3D ellipsoid volume changes (by 282–378%) result mainly from the TP load increase, especially for the long-term predictions of RCP4.5 and RCP8.5 scenarios (VS2 and VS4). Although that causes a decrease in the TN:TP ratio, the increase in chlorophyll “a” concentrations is still visible since this is related to the biomass transport from the lake. The range of ellipsoid changes is also similar in cluster C, covering exclusively the winter/spring months. However, in this case, the forecasted increase in temperatures possibly moderates nutrient delivery from the basin, bringing the TN:TP ratio into the more favorable eutrophication zone. Changes of nutrient load delivery for the basin are also explicit in cluster D, containing results both for extremely eutrophication unfavorable conditions for the upper Nielba River and the highly eutrophic Łękno Lake. However, here the impact of variant scenarios is different than in the other clusters. For the majority of scenarios (VS1–VS3), the decrease in TN loads is visible, most likely caused by the decrease in precipitation-induced runoff. In cluster B, ellipse/ellipsoid changes manifest themselves mostly in dispersion of its outputs, especially for the long-term RCP8.5 scenario (VS4, by 1198%). Since this cluster contains mostly summer/autumn modeling outputs from all the sub-basins, the detailed answer which factors control this process should be subjected to further multiparameter analysis including other parameters, not only nutrient loads. It should also be noted that extreme changes of ellipsoid values are mainly controlled by the outlier values, which mark mainly highly elevated chlorophyll “a” concentrations. This signifies model reaction depicting exceptionally favorable biomass production conditions, which could be induced by climate change in the future.

Our results show that future climate changes, expressed in terms of temperature and precipitation, will in general amplify the existing pattern of biomass production. For the riverine sections of the upper Nielba River, the chlorophyll “a” concentrations will be increased only during the winter/spring months when surface runoffs are already high and will be elevated in the future by the precipitation increase. As for the remaining months of the year, despite the favorable TN:TP ratios (between 9 and 50), the biomass production remains negligible due to the surface runoff decline and self-purification processes. Such a pattern is characteristic for many rivers with a similar hydrological regime.^104,105^ As for the lower part of the Nielba River, the present and future biomass production issues are controlled by the presence of lakes, wherein the intensity of future biomass production can be enhanced by the predicted increase in the nutrient load delivery. The TN:TP imbalances, and especially TP impact on biomass production should be further investigated. In general, the relationship between phosphorus and biomass seems to be closer for rivers than for lakes, and the phosphorus variability may result in a rapid river ecosystem reaction.^106,107^ Our mathematical approach, although limited to only three factors, shall be very beneficial for extensive database analysis and capturing relevant biomass production relationships. This approach allowed us for simultaneous analyses of nitrogen and phosphorus loads and chlorophyll “a” concentrations and showed that the increase in nutrients due to climate change does not necessarily lead to a parallel biomass development. However, our results clearly indicate that high chlorophyll “a” episodes will become more frequent and more dependent on the phosphorus changes than the nitrogen ones.

### 4.4 Future Research

Our findings are extremely important for land and water management in basins vulnerable to eutrophication. Since their identification and precise delineation is already possible,^108^ the next research step should be focused on elucidation of the other factors responsible for the biomass production potential within a river (e.g., radiation, soil properties, crops and fertilization, and hydrological features). Moreover, such multiparameter analysis could provide leads on extreme chlorophyll “a” concentrations, which as shown by the current study, can appear more and more frequently. In the future studies, special attention shall be also paid to the land use changes since in the case of biomass production, they are equally important as the climate ones. Also, the future modifications in farming activities and cultivation patterns following climate alterations should be taken into consideration. However, the latter would require more detailed information from the local/regional studies, which is still scarce. Also, the climate changes predictions require the utmost attention in terms of their reliability and spatial resolution. Despite many years of research, there is still a lack of reliable data on the local patterns of climate change, and climate adaptation is still lagging.^109^

## Supporting Information Available

Additional information on the analyzed catchment area (Ch1), CHLA module and construction (Ch2), and calibration, verification, and validation of the Macromodel DNS/SWAT (Ch3) and additional tables and figures of (Figure S1) land use and soil types in the sub-basin of the main stream of the Nielba River, (Figure S2) monthly distribution of flow rates (m^3^/s), TN and TP loads (kg/month), and TN:TP ratios in each sub-basin of the Nielba River, (Table 1) Macromodel DNS/SWAT input data, source, and resolution for the Wełna River basin area, (Table 2) classification of value ranges for statistical measures used during calibration, verification, and validation, and (Table 3) the Wełna River model calibration, verification, and validation results for daily simulations (PDF)
