# Supplementary material for: Biomass Production Potential in a River under Climate Change Scenarios
Source: Environ Sci Technol. 2021 Aug 3;55(16):11113–24. doi: 10.1021/acs.est.1c03211 (PMC8384234; doi:10.1021/acs.est.1c03211)
Supplement: Supplementary file 1 — es1c03211_si_001.pdf [file es1c03211_si_001.pdf]

Supporting Information for:

Biomass production potential in a river under climate change scenarios

Paulina Orlińska-Woźniak[1] , Ewa Szalińska[2], Ewa Jakusik[1], Damian Bojanowski[2], Paweł Wilk[1]

[1] Institute of Meteorology and Water Management, National Research Institute, Podleśna 61, 01-673 Warsaw, Poland

[2] Faculty of Geology, Geophysics and Environmental Protection, AGH University of Science and Technology, A. Mickiewicza Av. 30, 30-059 Krakow, Poland

Corresponding Author:
Ewa Szalińska - Faculty of Geology, Geophysics and Environmental Protection, AGH University of Science and Technology, A. Mickiewicza Av. 30, 30-059 Krakow, Poland. https://orcid.org/0000-0002-5483-193X
Email: eszalinska@agh.edu.pl

Chapter:

Ch1. Study Area 2
Ch2. Water Quality.. 3
Ch3. Chlorophyll ‘a’ Assessments 3
Ch3. Modeling Tool 5
Ch4. Graph for Monthly Distribution of Flow and Nutrients Simulation Results 8

Figure S 1. Land use and soil types in the sub-basin of the main stream of the Nielba River. 2
Figure S 2. Monthly distribution of flow rate (m3/s), TN and TP loads (kg/month), and TN:TP ratio in each sub-basin of the Nielba River.. 8

Ch1. Study Area

The Nielba River has been divided into 7 sub-basins with three flow-through in its lower part:

- Łękno Lake (area 75.30 ha, average depth 2.8 m) is characterized by a very strong eutrophication (hypertrophy) status [1], which leads to a gradual overgrowing of the lake. The main source of pollution is attributed to the local wastewater treatment plant which discharges effluents with a high content of nutrients [2].
- Bracholińskie Lake (area 64.72 ha, average depth 2.6 m) is a small water body, which in 2006 experienced an ecological disaster caused by a transfer of residues from the alcohol production facility to the lake's waters [3]. Since then, reclamation works have been carried out. Currently, the lake waters are classified as strongly eutrophic, based on the state monitoring results [4].
- Rgielskie Lake (area 156.36 ha, average depth 5.3 m) constitutes the largest lake on the main watercourse and it is considered the cleanest one. Nevertheless, its water quality according to the monitoring results are classified as below good [4].

The Nielba River basin has a typically lowland and agricultural character, where the afforestation level (FRST) is about 9%, and the urbanized areas (URBN) cover slightly more than 5% of the area (Figure S 1). The remaining area (82%) of the basin area is covered by arable land (AGRO). Only the last section of the river (sub-basin 76) differs from the others due to the 38% plus share of urbanized area (city of Wągrowiec). Also characteristic to this area, is the dominance of sandy soils, occupying on average more than 88% of the basin area. In addition, there is a small percentage of loess soils, light loam, and peat-mud-muck soils present (Figure S 1).

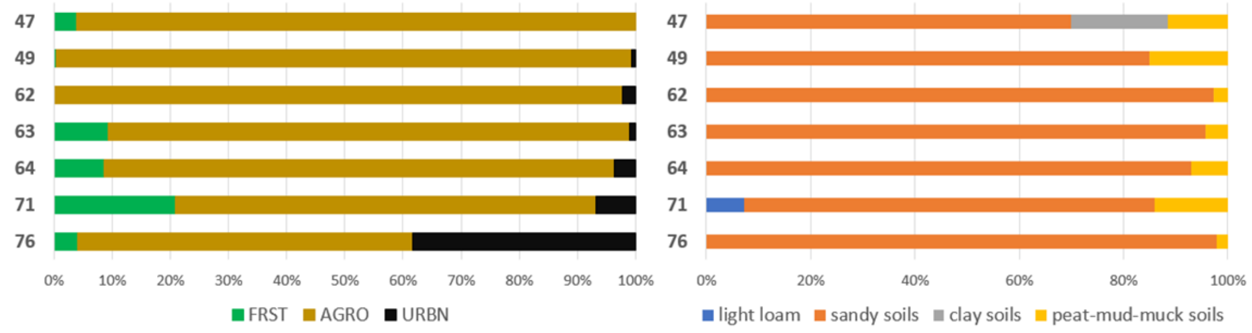

Figure S 1. Land use and soil types in the sub-basin of the main stream of the Nielba River.

In this part of Poland polar-sea air masses prevail, which make summers cooler and winters milder than in the eastern, more continental parts of the country. West winds with a speed of 2.5 m/s to 3.5 m/s dominate [5-6]. The average annual temperature is around 8.2°C, and in the case of individual seasons: 0.2°C - winter (Dec-Feb), 9.3°C - spring (Mar-May), 19.1°C - summer (Jun-Aug), and 9.7°C - autumn (Sep-Nov). The growing season is one of the longest in Poland, beginning at the end of March and lasting about 220 days. The annual rainfall here is between 500 and 550 mm, and snow cover occurs for a maximum of about 51–57 days [5-7]. The analyzed basin area is one of the driest regions in Poland, where the phenomenon of drought caused by a deficit of precipitation occurs with an increasing frequency [8-9] forcing changes in fertilization methods and negatively affecting the development of plants [10-12]. The flows on the Nielba River are low and fluctuate significantly (0.78 to 2.05 m3/s), which is strongly related to the meteorological situation in the basin area [13-14]. The analyzed river is characterized by a snow-rainy hydrological regime [15-16]. After reaching the spring maximum (January to April), river flow decreases. In summer (July to August) the water flow is much lower than in spring. The minimum period is generally from July to October.

Ch2. Water Quality

The Baltic Sea is struggling with an increasingly intense problem of eutrophication. The basin of the Odra estuary (which is in the hydrological continuum of the Nielba River) usually exceeds the Helcom threshold for chlorophyll a twice and has a growing tendency. The rivers flowing into the Baltic Sea in this region have a great potential to intensify eutrophication due to their biogenic quality. In particular sections of the river course, conditions favoring inland eutrophication appear as well [17]. The CHLA content standards for river waters constituting the algal bloom are usually between 15 and 30 µg / l [18] [19]. Such values often appear in the Odra catchment, which is both a local problem and a problem for the Baltic drainage area.

The catchment area of the Nielba River, as a tributary of the Wełna river, is defined as vulnerable to eutrophication. In Nielba, water quality standards are exceeded with regard to nitrogen and total phosphorus as well as easily decomposable organic compounds [20] High concentrations of CHLA appear in less shaded sections and those associated with flow lakes. Seasonal fluctuations in water quality and changes in phytoplankton pressure throughout the growing season are noted. Variation is also observed in individual sections, which is related to the variability of the temperature and oxygenation of the waters.

Ch3. Chlorophyll ‘a’ Assessments

The analyzes were carried out using the SWAT model, which allows taking into account both the processes in the land phase and the river bed phase, influencing the amount of CHLA. In the land phase, land use and related data on soil chemistry and fertilization doses determining TN and TP loads are of major importance. When simulating surface runoff, the model also estimates the river nutrient uptake and their effect on biomass production using the relationship between the nutrient enrichment index (TN and TP), CHLA and algae growth potential expressed by the following equation (1):

$$(AGP + CHLA) * v_{surf} = f * \left(\frac{TN}{TP}\right)^{g} \quad (1)$$

Where *AGP* is algal growth potential, *CHLA* is chlorophyll ‘a’ concentration in surface runoff, v_{surf} is the surface runoff flow rate, *TN* is the total nitrogen load, *TP* is the total phosphorus load, and where f and g represent potential export of TN and TP from the land phase reaching the river.

The SWAT module simplifies this relationship by making the CHLA simulation dependent on the size of surface runoff and TN and TP are expressed by the following equations (2-4)

$$CHLA = 0 \; if \; (v_{surf} < 10^{-5} \; m^3/s) \; or \; (TP \; and \; TN < 10^{-6}) \quad (2)$$

$$CHLA = \frac{0.5*10^{2.7}}{v_{surf}} \; if \; (v_{surf} > 10^{-5} \; m^3/s) \; and \; (TP \; and \; TN > 10^{-6}) \quad (3)$$

$$CHLA = \frac{0.5*10^{0.5}}{v_{surf}}\ if\ (v_{surf} > 10^{-5}m^3/s), TP < 10^{-6}\ and\ TN > 10^{-6} \quad (4)$$

The SWAT model also simulates the nutrient processes in the river bed phase. The processes combine nutrient cycles, biological oxygen demand, and algal dynamics. Algae concentrations are influenced by environmental factors and nutrient supply. Therefore, the growth and decay of algae/CHLA are calculated as a function of: growth rate, respiration rate, settling rate, and amount of algae present in the stream. The change in algal biomass is expressed by following equation (5):

$$\Delta algae = \left((\mu_a * algae) - (\rho_a * algae) - \left(\frac{\sigma_1}{depth} * algae\right)\right) * TT \quad (5)$$

Where: *algae* is the change in algal biomass concentration, u_a is the local specific growth rate of algae, p_a is the local respiration or death rate of algae, $ó_1$ is the local settling rate for algae, *depth* is the depth of water in the channel, *algae* is the algal biomass concentration at the beginning of the day, and *TT* is the flow travel time in the reach segment.

SWAT first calculates the growth rate at 20°C and then adjusts the growth rate for water temperature. In the described studies, the multiplicative option was used, including growth factors for light, and nitrogen and phosphorus together to determine their net effect on the local algal growth rate as expressed by the following equation (6):

$$\mu_{a20} = \mu_{max} * FL * FN * FP \quad (6)$$

Where: u_{a20} is the local specific algal growth rate at 20°C, u_{max} is the maximum specific algal growth rate, *FL* is the algal growth attenuation factor for light, *FN* is the algal growth limitation factor for nitrogen, and *FP* is the algal growth limitation factor for phosphorus.

The local respiration or death rate of algae represents the net effect of three processes: the endogenous respiration of algae, the conversion of algal phosphorus to organic phosphorus and the conversion of algal nitrogen to organic nitrogen. The respiration rate is adjusted to the local water temperature using the following relationship (7):

$$\rho_a = \rho_{a,20} * 1.047^{(Twater-20)} \quad (7)$$

Where: p_a is the local respiration rate of algae, $p_{a,20}$ is the local algal respiration rate at 20°C, *Twater* is the average water temperature for the daily hours.

The local settling rate of algae represents the net removal to algae due to settling. The settling rate is adjusted to the local water temperature using the following relationship (8):

$$\sigma = \sigma_{1.20} * 1.024^{(Twater-20)} \quad (8)$$

Where ð is the local settling rate of algae, and $ð_{1.20}$ is the local algal settling rate at 20oC

Ch3. Modeling Tool

The SWAT model for the Wełna River basin, commissioned by the State Water Holding - Polish Waters (SWH-WP) under the project “Identification of pressures in water regions and river basin districts” [21], was adapted for the current study, and then used as part of the Macromodel DNS/SWAT (Discharge-Nutrient-Sea/Soil Water Assessment Tool). The Nielba River was separated from the Wełna River model based on the boundaries of the sub-basin. The Macromodel DNS/SWAT is a tool developed at the Institute of Meteorology and Water Management - National Research Institute (IMGW-PIB) and has already been described in detail in numerous publications [22-27]. The Macromodel DNS/SWAT for the study area has been created using the following data presented in Table S 1.

Table S 1. Macromodel DNS/SWAT input data, source and resolution for the Wełna River basin area.

Description	Data source	Data resolution
Map of Poland hydrographical divisions	State Water Holding-Polish Waters, https://isok.gov.pl/hydroportal.html	5m, 1:10000
Digital elevation model (DEM)	Nation Protection IT System, https://isok.gov.pl/index.html	10m, 1:20000
Cross-sections of river channels	Nation Protection IT System - https://isok.gov.pl/hydroportal.html	-
Land use map (development of the river banks)	Corine Land Cover (CLC 2012), images from the Landsat 8 satellite http://clc.gios.gov.pl/index.php/26-clc-2012, https://bdl.stat.gov.pl/BDL	10 m
Soil map	Institute of Soil Science and Plant Cultivation, forest soil maps	2.5m, 1:100 000, 1: 10 000
Meteorological data - air temperature, precipitation, humidity, wind speed and total radiation	Institute of Meteorology and Water Management - National Research Institute - https://danepubliczne.imgw.pl/	for 250 stations located directly in the basin, and within 20 km from its borders
Drained areas - average depth of drains, average drainage time	Chief Inspectorate for Environmental Protection - https://www.gios.gov.pl/pl/stan-srodowiska/monitoring-wod	-
Point sources of wastewater, population not connected to the sewage system and fish ponds	National Program for Urban Waste Water Treatment - https://www.wody.gov.pl/nasze-dzialania/krajowy-program-oczyszczania-sciekow-komunalnych, Central Statistical Office - https://bdl.stat.gov.pl/BDL, State Water Holding-Polish Waters	-
Agrotechnical treatments, livestock breeding, the amount of mineral and natural fertilizers	Central Statistical Office - https://bdl.stat.gov.pl/BDL/	-
Atmospheric deposition, water erosion and geochemical background	Chief Inspectorate for Environmental Protection - http://powietrze.gios.gov.pl/pjp/archives	-

The Macromodel for the Welna River was calibrated, verified, and validated to capture both aspects of simulation, quantitative (flow) and qualitative (TN, TP, and sediment). The choice of the time periods and calculation profiles was imposed by availability of the monitoring data. For the quantitative part of the Macromodel, the daily flow data (source: IMGW-PIB) for the period of 18 years (2001-2018) from the gauging stations at the Welna River (Pruśce and Kowanówko) and its tributary (Flinta River - Ryczywół) were used (Figure 1 of Manuscript). The data for the examination of the qualitative part of the Macromodel (TN, TP, sediment, and CHLA) originated from the State Monitoring System stations localised at the Welna River (Oborniki and Rogoźno) and covered a period of 15 years (2001-2015). The first three years (2001-2003) were used to condition the model (i.e., warm-up or run-up for the SWAT simulations) [28], and the subsequent period for calibration (2004-2011), verification (2012-2018 - quantitative model, and 2012-2015 - qualitative model) and validation (2004-2015).
Calibration of the model was performed using the SWAT-CUP program developed by [29] and the SUFI-2 algorithm. Sensitivity analysis performed with the Latin Hypercube One-factor-at-a-Time (LH-OAT) sampling approach was used to identify the most influential estimation of constituent load (calibration) [30-32]. Then, a formulated regression model was used to estimate loads over a user-specified time interval (estimation).

Three statistical measures, coefficient of determination (R2) [33], percent bias (PBIAS) [34], and Kling-Gupta efficiency (KGE) [35-36], have been used to indicate the Welna River model performance, with the value ranges presented in Table S 2. For the quantitative part of the model (flow), the calibration and verification coefficients R2 and KGE were in the range of 0.66-0.90 (Table S 3), which classified the model performance generally as good and very good for the main river (Wełna), and satisfactory and good for its tributary (Flinta). The similar model performance was indicated by the PBIAS values (2-10%). Lower performance of the model for the Flinta River (R2) was attributed to the lower frequency of the flow data. During the validation procedure all the coefficient values rated the model performance for daily flow simulations as very good.

As for the qualitative part of the model, the model performance for TN simulations can be considered as very good and good, according to the all-applied coefficients (0.51-0.90 and 12-20%, for R2/KGE and PBIAS, respectively). Lower model performance, mostly satisfactory, was however observed for TP. In this case, the different sources of phosphorus than nitrogen compounds in the basin have to be considered (surface vs. point sources), and variability of the TP temporal distribution patterns which frequently affects the quality of TP simulations [37-39]. As for the sediment calibration and validation, the obtained results rated the model's performance as very good and good (0.52-0.81 and 20-22% for R2/KGE and PBIAS, respectively). The CHLA calibration and validation was performed on the basis of monitoring data from the calculation profile of the Wełna river in Oborniki and Rogoźno. The R2 was used to compare the observed and simulated values, which obtained the value of 0.52 and 0.48, respectively (Table S3).

The calibrated, verified, and validated Macromodel for the Wełna River basin was considered a reliable basis for subsequent calculations performed at any selected part of this area. These calculations, limited to the boundaries of the Nielba River basin, served as the baseline scenario in the subsequent variant scenario analysis. Since, a good practice requires to use simulation results only for the period the model has been at least verified, therefore, only years 2005-2007 have been used for the subsequent analyses. This choice was dictated by the availability of the chlorophyll 'a' monitoring data. Since this period includes a wet, medium and relatively dry year, therefore the data obtained for these years can be considered as representative.

Table S 2. Classification of value ranges for statistical measures used during calibration, verification, and validation, based on [22] and [40-42].

Performance Rating	R^2			PBIAS % (+/-)			KGE
	flow	TN	sediment/TP	flow	TN/TP	sediment	flow/TN/TP/sediment
very good	> 0.85	> 0.7	> 0.8	< 5	< 15	< 20	>0.75
good	0.75<R2<0.85	0.6<R2<0.7	0.65<R2<0.8	5<PBIAS<10	15<PBIAS<20	20<PBIAS<40	0.5 - 0.75
satisfactory	0.6<R2<0.75	0.3<R2<0.6	0.40<R2<0.65	10<PBIAS<15	20<PBIAS<30	40<PBIAS<70	0 - 0.5
unsatisfactory	< 0.6	< 0.3	< 0.4	> 15	> 30	> 70	<0

Table S 3. The Wełna River model calibration, verification and validation results for daily simulations.

Calculation profile	Parameter	R^2	PBIAS	KGE
	Calibration			
Flinta - Ryczywol	flow	0.71	-8	0.78
Wełna - Prusce		0.85	-5	0.9
	TN	0.86	-20	0.7
Wełna - Oborniki	TP	0.60	30	0.53
	sediment	0.81	-22	0.65
	CHLA	0.52	-	-
	Verification			
Flinta - Ryczywol	flow	0.66	2	0.62
Wełna - Prusce		0.83	10	0.86
Wełna - Oborniki	TN	0.86	12.5	0.67
	TP	0.38	-25	0.21
	Validation			
Wełna - Kowanowko	flow	0.8	15	0.81
	TN	0.9	-14	0.51
Wełna - Rogoźno	TP	0.35	23	0.47
	sediment	0.77	-20	0.52
	CHLA	0.48	-	-

Ch4. Graph for Monthly Distribution of Flow and Nutrients Simulation Results

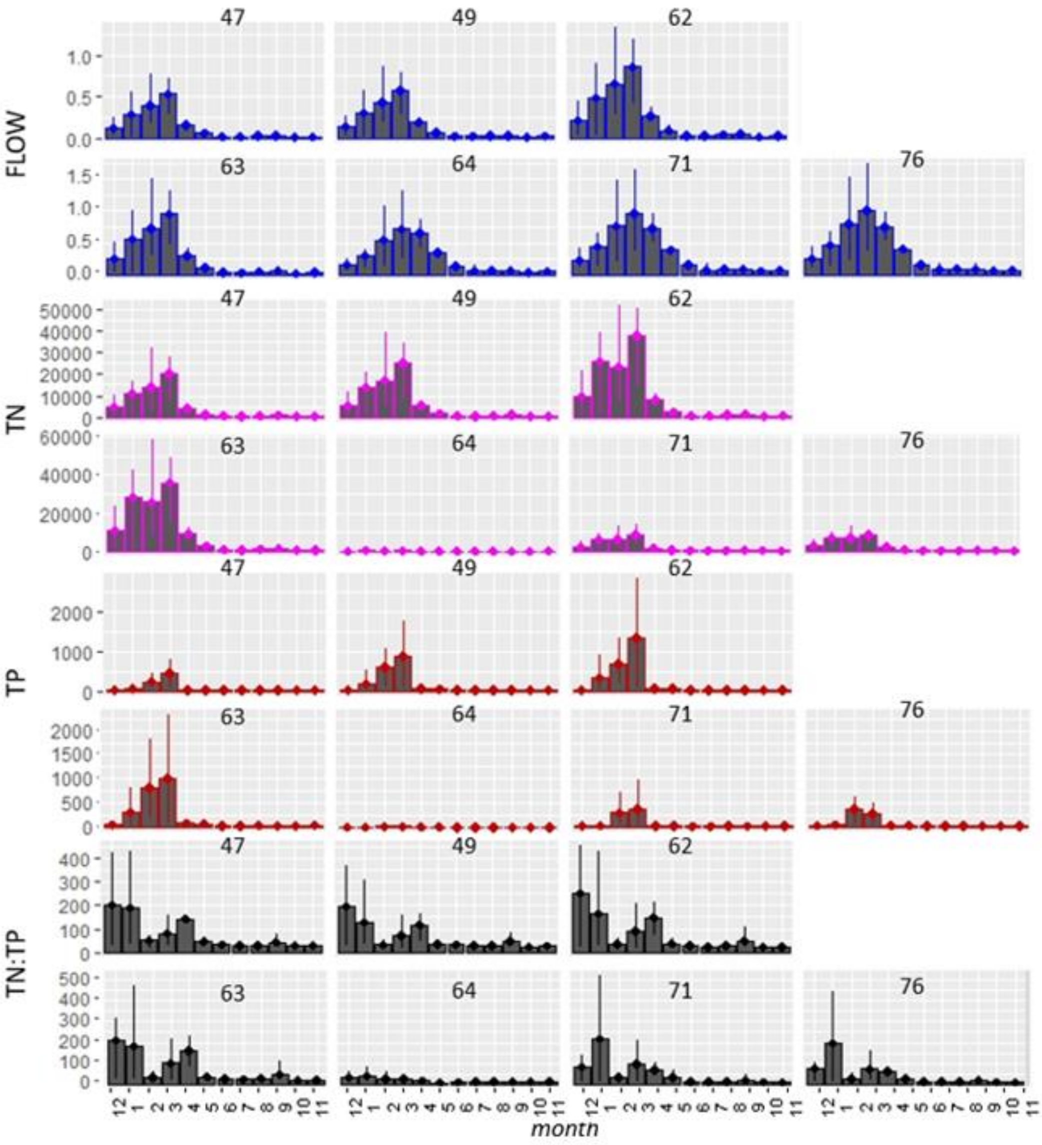

Figure S 2. Monthly distribution of flow rate (m3/s), TN and TP loads (kg/month), and TN:TP ratio in each sub-basin of the Nielba River.

References

[1] Dondajewska, R., Gołdyn, R., Kowalczewska-Madura, K., Kozak, A., Romanowicz-Brzozowska, W., Rosińska, J., Budzyńska, A., Podsiadłowski, S. (2020). Hypertrophic lakes and the results of their restoration in Western Poland. In Polish River Basins and Lakes–Part II (pp. 373-399).https://doi.org/10.1007/978-3-030-12139-6_17 .

[2] Dondajewska, R., Gołdyn, R., Messyasz, B., Kowalczewska-Madura, K., Cerbin, S. (2019). A shallow lake in an agricultural landscape–water quality, nutrient loads, future management. Limnological Review, 19(1), 25-35. 10.2478/limre-2019-0003 .

[3] Budzyńska, A., Kozak, A., Gołdyn, R. (2019) Toxic cyanobacterial blooms in lakes and reservoirs in Wielkopolska region (western Poland), Water Ecosystems 45. (in Polish)

[4] GIOŚ, (2020), Chief Inspectorate for Environmental Protection. Synthetic report on the classification and assessment of the state of surface water bodies for 2019, based on data from 2014-2019. https://www.gios.gov.pl/pl/stan-srodowiska/monitoring-wod (in Polish) (accessed 14.04.2021) .

[5] EPP (2016). Environmental protection program for the Wielkopolskie Voivodeship for 2016-2020, https://bip.umww.pl/artykuly/2822130/pliki/20161003123028_programochronyrodowiskadlawojewdztwawielkopolskiegonalata20162020.pdf (accessed 14.04.2021).

[6] Szyga-Pluta, K. (2018). Temporal and spatial variability of atmospheric precipitation in Wielkopolska in the 1981–2014 period. Polish Geographical Review, 90(3). (in Polish)

[7] Szwed, M. (2017). Projections of changes of areal evapotranspiration for different land-use units in the Wielkopolska Region (Poland). Theor. Appl. Climatol., 130(1-2), 291-304. https://doi.org/10.1007/s00704-016-1880-0 .

[8] Łabędzki, L. Ł., Bąk, B. (2017). Impact of meteorological drought on crop water deficit and crop yield reduction in Polish agriculture. Journal of Water and Land Development, 34(1), 181-190. 10.1515/jwld-2017-0052

[9] Kubicz, J., Bąk, B. (2019). The Reaction of Groundwater to Several Months' Meteorological Drought in Poland. Pol. J. Environ. Stud., 28(1). https://doi.org/10.15244/pjoes/81691

[10] Yu, C., Li, C., Xin, Q., Chen, H., Zhang, J., Zhang, F., Li, X., Clinton, N., Huang, X., Yue, Y., Gong, P. (2014). Dynamic assessment of the impact of drought on agricultural yield and scale-dependent return periods over large geographic regions. Environ Model Softw, 62, 454-464. https://doi.org/10.1016/j.envsoft.2014.08.004.

[11] Saravia, D., Farfán-Vignolo, E. R., Gutiérrez, R., De Mendiburu, F., Schafleitner, R., Bonierbale, M., Khan, M. A. (2016). Yield and physiological response of potatoes indicate different strategies to cope with drought stress and nitrogen fertilization. Am. J. Potato Res., 93(3), 288-295. https://doi.org/10.1007/s12230-016-9505-9.

[12] Siebert, J., Sünnemann, M., Auge, H., Berger, S., Cesarz, S., Ciobanu, M., Guerrero-Ramírez, R. N., Eisenhauer, N. (2019). The effects of drought and nutrient addition on soil organisms vary across taxonomic groups, but are constant across seasons. Scientific reports, 9(1), 1-12. https://doi.org/10.1038/s41598-018-36777-3.

[13] Messyasz, B., Szczuka, E., Kaznowski, A., Burchardt, L. (2010). The spatial changes of phytoseston and microbiological parameters in lowland rivers during the summer period. Pol. J. Environ. Stud., 19(6), 1269-1277.

[14] Fabrowska, J., Messyasz, B., Pankiewicz, R., Wilińska, P., Łęska, B. (2018). Seasonal differences in the content of phenols and pigments in thalli of freshwater Cladophora glomerata and its habitat. Water research, 135, 66-74. https://doi.org/10.1016/j.watres.2018.02.020.

[15] Kanclerz, J., Murat-Błażejewska, S., Janicka, E., Adamska, A. (2018). Environmental flows of lowland rivers with disturbed hydrological regime on the example of Mała Wełna River. Annual Set The Environment Protection, 20. .
[16] Wrzesiński, D., Perz, A. (2016). Features of the river runoff regime in the Warta catchment area. 10.14746/bfg.2016.7.21 (in Polish)
[17] Background information on the Baltic Sea catchment area for the Sixth Baltic Sea Pollution load compilation (PLC-6) 2020 Baltic Marine Environment Protection Commission, HELCOM.
[18] Climate change and eutrophication risk thresholds in English rivers, Report – SC140013/R2, Environment Agency.
[19] Chlorophyll in transitional, coastal and marine waters, INDICATOR ASSESSMENT, HELCOM raport
[20] Joniak, T., Rybak, M., & Sprawka, M. (2014). Ocena przestrzennego zróżnicowania właściwości fizyczno-chemicznych wody w rzekach Wełnie i Flincie. Stan jakościowy wód a potencjalne i rzeczywiste źródła zanieczyszczeń. W: J. Bator, M. Gąbka, E. Jakubas (red.), Koncepcja lasu modelowego w zarządzaniu i ochronie różnorodności biologicznej rzek Wełny i Flinty (Wielkopolska). Bogucki Wyd. Naukowe, Poznań, 43-56..
[21] SWH-PW, 2020. State Water Holding - Polish Waters. Identification of pressures in water regions and river basin districts. https://www.apgw.gov.pl/pl/III-cykl-prace-realizowane-w-cyklu (accessed 20.07.2020)
[22] Orlińska-Woźniak, P., Szalińska, E.,Wilk, P., 2020a. Do land use changes balance out sediment yields under climate change predictions on the Sub-Basin scale? The Carpathian Basin as an example. Water 12 (5), 1499. https://doi.org/10.3390/w12051499.
[23] Wilk, P., Orlińska-Woźniak, P., Gębala, J., Ostojski,M., (2017). The flattening phenomenon in a seasonal variability analysis of the total nitrogen loads in river waters. Technical Transactions (11), 137–159.
[24] Wilk, P., Orlińska-Woźniak, P., Gębala, J., (2018)a. The river absorption capacity determination as a tool to evaluate state of surface water. Hydrol. Earth Sys. Sci. 22 (2), 1033–1050. https://doi.org/10.5194/hess-22-1033-2018.
[25] Wilk, P., Orlińska-Woźniak, P., Gębala, J., (2018)b. Mathematical description of a river absorption capacity on the example of the middle Warta catchment. Environ. Prot. Eng. 44 (4), 99–116. https://doi.org/10.5277/epe180407.
[26] Szalińska, E., Wilk, P., 2018. Sediment quantity management in polish catchment-riversea systems–should we care? Economics and Environment 3 (66), 25–37.
[27] Orlińska-Woźniak, P., Wilk, P., Szalińska, E., 2020b. Delimitation of nutrient vulnerable zones-a comprehensive method to manage a persistent problem of agriculture. Agric. Syst. 83, 102858. https://doi.org/10.1016/j.agsy.2020.102858
[28] Niraula, R., Meixner, T., Norman, L. M. (2015). Determining the importance of model calibration for forecasting absolute/relative changes in streamflow from LULC and climate changes. J. Hydrol., 522, 439-451. https://doi.org/10.1016/j.jhydrol.2015.01.007
[29] Abbaspour, K.C.; Rouholahnejad, E.; Vaghefi, S.R.; Srinivasan, R.; Yang, H.; Kløve, B. A. (2015) continental-scale hydrology and water quality model for Europe: Calibration and uncertainty of a high- resolution large-scale SWAT model. J. Hydrol. 524, 733–752..
[30] Park, G.A.; Park, J.Y.; Joh, H.K.; Lee, J.W.; Ahn, S.R.; Kim, S.J. (2014) Evaluation of mixed forest evapotranspiration and soil moisture using measured and swat simulated results in a hillslope watershed. KSCE J. Civ. Eng. 18, 315–322. https://doi.org/10.1007/s12205-014-0193-z.
[31] Park, Y., Cho, K. H., Park, J., Cha, S. M., Kim, J. H. (2015). Development of early-warning protocol for predicting chlorophyll-a concentration using machine learning models in freshwater

and estuarine reservoirs, Korea. Sci. Total Environ., 502, 31-41. https://doi.org/10.1016/j.scitotenv.2014.09.005.

[32] Khoi, D.N., Thom, V.T. (2015). Parameter uncertainty analysis for simulating streamflow in a river catchment of Vietnam. Glob. Ecol. Conserv. 4, 538–548.https://doi.org/10.1016/j.gecco.2015.10.007

[33] Zhang, D. (2017). A coefficient of determination for generalized linear models. The American Statistician, 71(4), 310-316. https://doi.org/10.1080/00031305.2016.1256839

[34] Goshime, D. W., Absi, R., Ledésert, B. (2019). Evaluation and bias correction of CHIRP rainfall estimate for rainfall-runoff simulation over Lake Ziway watershed, Ethiopia. Hydrology, 6(3), 68. https://doi.org/10.3390/hydrology6030068

[35] Pool, S., Vis, M., Seibert, J. (2018). Evaluating model performance: towards a non-parametric variant of the Kling-Gupta efficiency. Hydrol. Sci. J. 63(13-14), 1941-1953. https://doi.org/10.1080/02626667.2018.1552002

[36] Knoben, W. J., Freer, J. E., Woods, R. A. (2019). Inherent benchmark or not? Comparing Nash–Sutcliffe and Kling–Gupta efficiency scores. Hydrology and Earth System Sciences, 23(10), 4323-4331. https://doi.org/10.5194/hess-23-4323-2019

[37] Lu, S., Kronvang, B., Audet, J., Trolle, D., Andersen, H. E., Thodsen, H., van Griensven, A. (2015). Modelling sediment and total phosphorus export from a lowland catchment: Comparing sediment routing methods. Hydrological processes, 29(2), 280-294. https://doi.org/10.1002/hyp.10149.

[38] Ostojski, M. S., Gębala, J., Orlińska-Woźniak, P., Wilk, P. (2016). Implementation of robust statistics in the calibration, verification and validation step of model evaluation to better reflect processes concerning total phosphorus load occurring in the catchment. Ecological modelling, 332, 83-93. https://doi.org/10.1016/j.ecolmodel.2016.04.004.

[39] Bauwe, A., Eckhardt, K. U., Lennartz, B. (2019). Predicting dissolved reactive phosphorus in tile-drained catchments using a modified SWAT model. Ecohydrology & Hydrobiology, 19(2), 198-209. https://doi.org/10.1016/j.ecohyd.2019.03.003

[40] Moriasi, D. N., Gitau, M. W., Pai, N., Daggupati, P. (2015). Hydrologic and water quality models: Performance measures and evaluation criteria. Transactions of the ASABE, 58(6), 1763-1785. 10.13031/trans.58.10715

[41] Patil, S. D., Stieglitz, M. (2015). Comparing spatial and temporal transferability of hydrological model parameters. J. Hydrol., 525, 409-417. https://doi.org/10.1016/j.jhydrol.2015.04.003

[42] Libera, D. A., Sankarasubramanian, A. (2018). Multivariate bias corrections of mechanistic water quality model predictions. J. Hydrol., 564, 529-541.https://doi.org/10.1016/j.jhydrol.2018.07.043
